# Systems approach to investigating host-pathogen interactions in infections with the biothreat agent *Francisella*. Constraints-based model of *Francisella tularensis*

**DOI:** 10.1186/1752-0509-4-118

**Published:** 2010-08-23

**Authors:** Anu Raghunathan, Sookil Shin, Simon Daefler

**Affiliations:** 1 Mount Sinai School of Medicine, Department of Medicine, One Gustave L. Levy Place, New York, NY 10029, USA; 2 National Chemical Laboratory, Pune, 411008, India

## Abstract

**Background:**

*Francisella tularensis *is a prototypic example of a pathogen for which few experimental datasets exist, but for which copious high-throughout data are becoming available because of its re-emerging significance as biothreat agent. The virulence of *Francisella tularensis *depends on its growth capabilities within a defined environmental niche of the host cell.

**Results:**

We reconstructed the metabolism of *Francisella *as a stoichiometric matrix. This systems biology approach demonstrated that changes in carbohydrate utilization and amino acid metabolism play a pivotal role in growth, acid resistance, and energy homeostasis during infection with *Francisella*. We also show how varying the expression of certain metabolic genes in different environments efficiently controls the metabolic capacity of *F. tularensis*. Selective gene-expression analysis showed modulation of sugar catabolism by switching from oxidative metabolism (TCA cycle) in the initial stages of infection to fatty acid oxidation and gluconeogenesis later on. Computational analysis with constraints derived from experimental data revealed a limited set of metabolic genes that are operational during infection.

**Conclusions:**

This integrated systems approach provides an important tool to understand the pathogenesis of an ill-characterized biothreat agent and to identify potential novel drug targets when rapid target identification is required should such microbes be intentionally released or become epidemic.

## Background

Emerging or re-emerging infectious agents are rarely subject of extensive preceding experimental investigations by sheer implication of their definition, as they would, for example, exist for model pathogens such as *Salmonella*. Even when their potential for global pandemics or for being used as biothreat agents is recognized, in-depth data cannot be easily generated in a timely fashion. While high-throughput data such as full genome sequences, microarray gene expression profiles, or proteomic data can be obtained quite efficiently for the pathogen in question, the context for proper interpretation of such data is often missing. One such case is represented by *Francisella tularensis*, the causative agent of tularemia [1]. This pathogen is highly infectious and can cause fatal systemic disease after inhalation of as little as 10 organisms. This extremely low infectious dose, ease of transmission via the aerosol route, and previous attempts to weaponize this microbe have led to its recognition as a biothreat agent [2]. Extensive research over the last years in the field of tularemia after the full potential of *Francisella *had been recognized has been focused on vaccine development, virulence factors, and whole genomic sequencing of *Francisella *isolates. There are few data sets for the physiological characterization of this bacterium during infection. Its central metabolic pathways still remain largely uncharacterized. However, growth of *Francisella *within macrophages or other potential host cells depends on its ability to utilize available nutrients and exploit this niche by presumably having to adjust its metabolism [1]. This is critical for the success of *Francisella *as an intracellular pathogen. At the same time, critical bacterial metabolic pathways represent the most likely targets for novel antibacterial strategies. In this setting computational systems biology approaches may facilitate the integration of high-throughput data, exclude redundant possibilities, and suggest novel hypotheses.

Constraints-based systems analysis after reconstruction of genome scale metabolic networks has emerged as a suitable tool for such tasks [3]. This type of reconstruction allows the evaluation of metabolic networks through flux balance and variability analysis, *in silico *gene deletion analysis, robustness analysis, and the successive application of other suitable constraints. Such analysis has already been implemented for organisms from all the three classes (archae, bacteria and eukaryotes) including *E. coli *[4], *Salmonella *[5], *Haemophilus *[6], *Saccharomyces *[7], and *Leishmania *[8]. For these organisms, large traditional and high-throughput experimental datasets were available. Here we employ this approach for *Francisella tularensis *with the goal of providing a framework for the integration of existing high-throughput data that might be used for interrogating pathogenesis and for identifying novel antibacterial targets.

## Results

### Reconstruction and analysis of the metabolic network of *F. tularensis*

**Figure 1:**
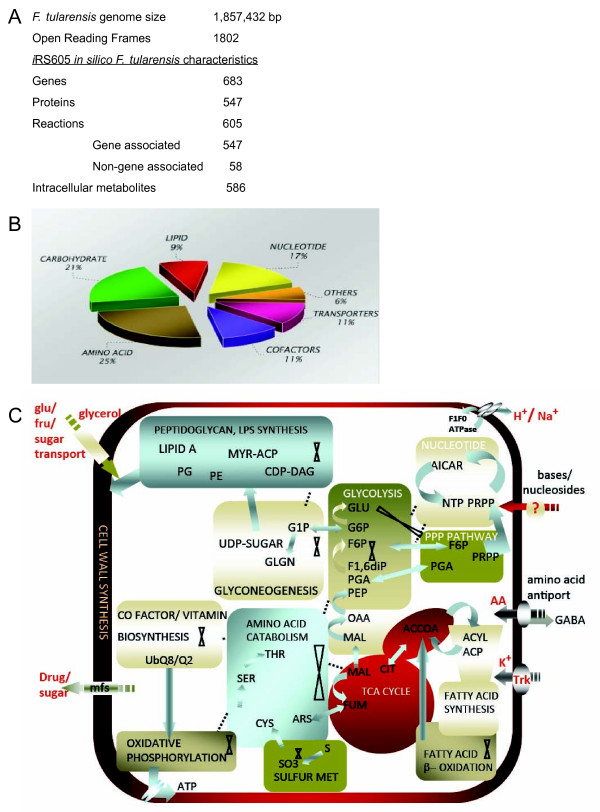
**Reconstruction characteristics for *i*RS605. A. Statistics of the *Francisella tularensis *subspecies *holarctica *vaccine strain (*F. tularensis *LVS) genome and its in silico reconstruction *i*RS605**. The in silico representation includes gene-protein-reaction relationships including isozymes, multimeric protein complexes, and multi-reaction catalyzing enzymes as described in the text. B. Functional classification of genes that are included in the model. C. Overview of metabolism in *Francisella*. Significant interconnections between pathways are shown. Pathways with missing genes are indicated by cross symbols.

The reconstruction process for building a metabolic network for *Francisella tularensis *subspecies *holarctica *vaccine strain (*F. tularensis *LVS) is described in detail in Material and Methods and followed previously described procedures [9,3]. The network reconstruction statistics for *i*RS605, which has 683 genes, 547 proteins, and 605 intra-system reactions, are summarized in Figure 1A. Throughout this manuscript *i*RS605 refers to the metabolic reconstruction of *Francisella *LVS. The low number of reactions in the reconstruction as compared to similar models like *E. coli *and *Salmonella *is the result of a reduced set of metabolic pathways in this organism and of limited legacy data to confirm the annotation of its genome. The reconstruction includes 481 single gene protein reaction relationships (GPR) and 66 multigene GPRs (Additional File S1). The genes included in the model represent those for which genomic, transcriptomic or physiological data exist in the literature in the form of gene-protein specific biochemistry or large-scale screens. Genes with high sequence homology to genes in other *Francisella *strains such as Schu4 or OSU118 are also included in the model. There are 55 non-gene associated reactions included in the model that lack experimental evidence but are essential for validation and prediction of a physiological phenotype *in silico *(Additional File S1C).

Gap analysis of the model identified several gaps in the network. There are gaps in a network when certain metabolites can only be consumed or produced. Since *Francisella *has several pathways that are disrupted or that miss genes [10], there are 102 dead end metabolites in the *Francisella *network (Additional File S2). The reactions catalyzed by these genes form a set of reactions whose presence or absence provide important hypotheses on possible connecting reactions that can be further experimentally investigated and can thus lead to efficient annotation of the genome. With this, a more functionally complete network can be obtained.

The distribution of pathway functions are summarized in Figure 1B. Twenty five percent of those constitute amino acid metabolism [10,3]. When these biosynthetic pathways are disrupted the respective compound is required as an essential component of a synthetic medium for growth. The partial amino acid pathways identified that are essential for growth [11,12] include those for Asp, Cys, Ser, Thr, Met, Tyr, Lys, Pro, Arg, His, Val, Ile, and Leu and comprise a significant part of the bacterial network.

Pathways identified to be incomplete by gap analysis and confirmed using gene sequence data are discussed below. Central metabolic pathways that are incomplete include the pentose phosphate pathway and glycolysis. The genome encodes all glycolytic genes but lacks the phosphofructokinase (PfkA) gene. Constraints based analysis suggests that the presence of fructose bisphosphatase (Fbp), allows *Francisella *to build up complex carbohydrates by gluconeogenesis. This suggests the use of the Embden-Meyerhof-Parnas (EMP) pathway for gluconeogenesis rather than glycolysis. The pathogen is then able to synthesize glucose from pyruvate, since it encodes phosphoenolpyruvate (PEP) carboxylase. This allows gluconeogenesis starting from oxaloacetate. Despite an incomplete glycolytic pathway and only an oxidative branch of the pentose phosphate pathway, genes predicted to encode sugar uptake systems might at least ensure partial energy generation via the oxidization of hexoses. The retention of transketolase and transaldolase genes of the nonoxidative branch of the pentose phosphate pathway indicates the ability to regenerate glucose-6-phosphate and oxidize hexoses to pyruvate, although without an energy yield. For energy generation, *Francisella *may thus prefer to oxidize amino acids or other organic compounds derived from the host. Thus network reconstruction and constraints-based analysis identify condition-dependent operational pathways that throw light on adjustments the organism is capable of making in different environments.

Pyrimidine and purine metabolism [13] and fatty acid biosynthesis in *Francisella *are virtually identical to those in *E.coli*. Several glycerol and phospholipid biosynthesis pathways are present in *Francisella *including phosphatidyl serine. However, there is no evidence for cardiolipin synthesis [14]. Orthologs of cardiolipin synthase are also absent. Diaminopimelic acid (DAP), which is an intermediate of lysine biosynthesis and an important compound in cell wall biosynthesis, cannot be synthesized, although lysine itself can be synthesized.

Although PAPS reductase is missing, *Francisella *is able to reduce sulfate to sulfide for macromolecule synthesis via the APS-PAPS pathway and eventually fix as cysteine. This may mean broader specificities of the enzymes involved and further emphasizes the need to test experimentally the predicted gaps in *Francisella *metabolism.

The majority of transport systems present in *Francisella *are secondary carriers [10]. The remaining transport systems are ABC-type carriers, consisting of a membrane-spanning permease and an ATP-binding subunit. Interestingly, periplasmic substrate-binding proteins, which usually are an integral part of such transport systems, are missing in most ABC-type carriers. Very few permeases that catalyze transport by a concentration gradient are found. In accordance with the advanced degeneration of its amino acid biosynthetic capability as discussed earlier, *Francisella *has retained several transport systems for amino acids [15], including LysE and SdaC, that are both secondary carriers for branched chain and hydroxy amino acids. There is also evidence for aquaglyceroporin GlpF that is involved in glycerol and water transport. It may also accept other small, uncharged organic molecules such as urea, glycine, or glycerolaldehyde as substrates. *Francisella *seems to posses uptake systems for several minerals and salts including a low and high affinity transport system for potassium [10,16], a secondary carrier for inorganic phosphate, and an antiport system for Na/H. *Francisella *also encodes an ABC transporter for manganese and zinc.

Figure 1C is a concise overview of *i*RS605 of the *Francisella *metabolic network. It emphasizes evidence of metabolic function for different pathways although many intermediate reactions are found missing. This does not reduce the significance of the pathway or reaction in the growth and survival of *Francisella*, and suggests dependence on the host for that particular function. *Francisella *seems to have kept redundant enzyme function to a minimum (lower number of isozymes) with metabolic analysis suggesting a broader specificity for enzymes.

### Metabolic functional states and *in silico growth of Francisella tularensis iRS605*

A constraints based flux balance model was derived from this metabolic reconstruction that can be used to predict growth both qualitatively and quantitatively under specified conditions. Flux balance analysis is set up as a linear optimization problem with maximization of the biomass function as the objective. This biomass objective function was defined by conducting experimental measurements for macromolecular composition of *F. tularensis*. The biomass objective function was defined using experimental data and literature for chemical composition studies of *F. tularensis *and includes metabolites in addition to experimentally determined growth associated ATP maintenance costs. We accounted for absence of phospholipids like cardiolipin that form integral parts of biomass in other organisms like *E.coli and Salmonella*. The reconstruction of the biomass function is critical to the success of the model in predicting physiological behaviors (equivalent to functional states of the network) since it defines the drain on several metabolites to make macromolecules that determine the biomass composition.

*i*RS605 was grown *in silico *by specifying appropriate exchange fluxes (Additional File S3) in defined *Chamberlain *media and using Flux Balance Analysis (FBA) to maximize the defined biomass function (described in detail in Methods). Calculated physiological states of *F. tularensis *indicate that amino acids can provide all the bulk carbon requirements for growth. Biomass production changed on varying amino acid concentration, which correlates with experimental data. Thirty-six percent of the total metabolic reactions were estimated to be operational (non zero flux value) during growth of *F. tularensis *on *Chamberlain*'s minimal media supplemented with glucose. This is higher than found in other bacteria such as *E. coli *and *Salmonella *[5] that use only 25% of their metabolic capacity to accomplish the same goal. Also, higher rates of ammonia exchange (up to 4 times) were predicted for *Francisella *than for *Salmonella*. Ammonia exchange was estimated using flux balance analysis. Amino acids were not only incorporated into biomass but also used for energy generation in *Francisella*. The molar fraction of the amino acids in biomass was experimentally determined (data not shown) and used to specify coefficients in the biomass equation. Different ATP requirements for growth and maintenance were thus incorporated in the *Francisella *model by combining specific growth rates, substrate uptake data, and flux balance analysis [17]. The accuracy of the model-predicted growth rates is dependent on this and was confirmed by comparing physiological growth rates on different substrates (as discussed below). Growth experiments and metabolic phenotyping for the pathogen in addition to existing literature were used for this purpose.

### Model *i*RS605 accurately predicts gene essentiality and virulence

*i*RS605 was used to perform *in silico *gene deletion studies that carry the potential to identify antibiotic drug targets. The model was initially used to determine condition dependent essential metabolic genes (lethal genes) needed for growth and survival. The conditions chosen were Chamberlain media with no additional carbon/energy source, Chamberlain media with six different primary carbon/energy sources (glucose, fructose, arabinose, ribose, xylose and glycerol), a rich media designed to simulate conditions found in macrophages, and complex Mueller-Hinton broth. Gene essentiality was assessed by deleting single reactions *in silico *and testing for growth. The essentiality of a given gene was determined by calculating the maximal growth rate using FBA when fluxes through associated reactions were constrained to be zero. This type of analysis identifies lethal gene deletions (where maximum growth rate is zero) and non-lethal gene deletions (where maximum growth rate is greater than zero. Isozymes and multi-unit enzymes and protein complexes are represented using Boolean logic and hence deletion of an enzyme subunit would force flux values through associated reactions to be zero, unless other isozymes are present. In environments where uptake reactions (available nutrients) are limited, more metabolic genes are essential than in the rich medium. Hence, gene deletion studies with minimal media predicted 25% more essential genes than in the case of enriched environments. These genes are condition-dependent lethals [18] and are based on the specific growth environment. Condition-independent lethal genes are those that are lethal irrespective of the environment. Unconditional essential genes were identified for *i*RS605 at the intersection of the sets of such essential genes in six environmental conditions including a simulated macrophage environment.

Based on this intersection set we found 105 condition independent lethal genes (Additional File S4). Given the scarcity of experimentally confirmed essential genes during infection with *Francisella*, we used data on virulence genes associated with metabolism to determine if our predictions matched any existing literature [19,23,22,35]. The identification of genes essential for survival not only suggests potential virulence genes, but is also important for understanding the minimal requirements for pathogen survival in the host cell. The model predicted essential genes involved in virulence with 47% accuracy (Table 1). Such single gene perturbation experiments are effective in identifying components in *Francisella *that are essential for the growth phenotype and whose deletion may cause attenuation in pathogens.

**Table 1:** Comparison of predicted *in silico *virulence with experimentally determined *in vivo *virulence

LVS locus	Schu4 locus	Gene	Virulence	
			***in silico***	***in vivo***

FTL_1071	FTT_1019c	guaA	+	+

FTL_1478	FTT_1317c	guaB	+	+

FTL_0395	FTT_0893	purM	+	+

FTL_0396	FTT_0894	purCD	+	+

FTL_1504	FTT_0721c	katG	-	+

FTL_1791	FTT_0068	sodB	-	+

FTL_0483	FTT_0413c	glgB	-	+

FTL_1262	FTT_0945		+	+

FTL_0838	FTT_1124	metN	+	+

FTL_0837	FTT_1125	metIQ	+	+

FTL_0789	FTT_1165c	aspC2	-	+

FTL_0606	FTT_1450	wbtM	-	+

FTL_0594	FTT_1462c	wbtC	-	+

FTL_0592	FTT_1464c	wbtA	-	+

FTL_0304	FTT_1490		-	+

FTL_1701	FTT_1631c	gplX	-	+

FTL_0058	FTT_1168		+	+

Accuracy of iRS635 virulence prediction = 47%

### Metabolic reactome during infection

The metabolic capacity of an organism is determined by all available alternate routes it can use to achieve its growth objective. As demonstrated for *Salmonella*, for a pathogen it is the ability to achieve this during infection [5] . Flux variability analysis (FVA) was used to identify such metabolic reactions that might be operational in different environments including that during infection. This allows us to identify the host metabolism that *Francisella *could exploit in order to survive and replicate.

In defined medium (Chamberlain with glucose) 32% of the network reactions that span a range of flux values when biomass production was optimal were operational. The constraining conditions that represent the environment during infection in the macrophage (Additional File S3) were derived by literature mining. Based on this nutrient composition, FVA identified only 103 reactions that can be utilized for growth via alternate routes. This suggests that only 6% of the whole genome (equivalent to 17% of the metabolic network in *i*RS605) spans a non-zero integer flux for optimal biomass production. This is less than half of that we found *in silico *for *Salmonella *iRR1083 in a similar environment [5]. *Francisella *potentially minimizes utilization of its own pathways during infection. The larger reactome (equivalent to 37% of *i*RS605) during optimal growth in a minimal medium (Chamberlain with glucose) supports this hypothesis. The condition-independent reactome for optimal growth identified 59 reactions (Additional File S5).

We compared the transcriptomic data from literature [36] and found that 33% of the genes are contained in the optimal reactomes under the same conditions *in silico *(Additional File S5). Proteomic data [37] identified 171 metabolic proteins by iTRAQ. 52% (89) of these proteins were detected *in silico *(Additional File S5). The prediction of metabolic capacity as a function of the reactome is lower for *Francisella *than for *Salmonella *[5] (77% ad 98% accuracy for transcriptomic and proteomic data respectively). A reduced metabolic genome increases dependency on availability of nutrients and necessary metabolites that cannot be made in the cell. The accurate representation of this environment is crucial in determining the predictive capability of *i*RS605. The lower predictive capability of the model in comparison to *Salmonella *is in part due to the incomplete knowledge of exchange fluxes that represent the environment during intracellular replication and infection and in part a result of *Francisella's *unique potential to exploit of the host niche for intracellular replication and growth.

### Effect of constraining metabolite flux and gene expression levels on growth

The effect of reducing expression levels of metabolic genes on growth was tested *in silico *using robustness analysis [38]. Pair-wise interrogation of growth as a function of varying expression of the metabolic genes (studied during infection) was conducted in different environments. Among the genes studied 25% of them were observed to have a differential effect on growth rate under *in vivo *and *in vitro *conditions. Robustness diagrams (RD) consist of trajectories describing the linear or non-linear relation between a specific metabolic enzyme concentration (correlated here to mRNA abundance) and growth.

**Figure 2:**
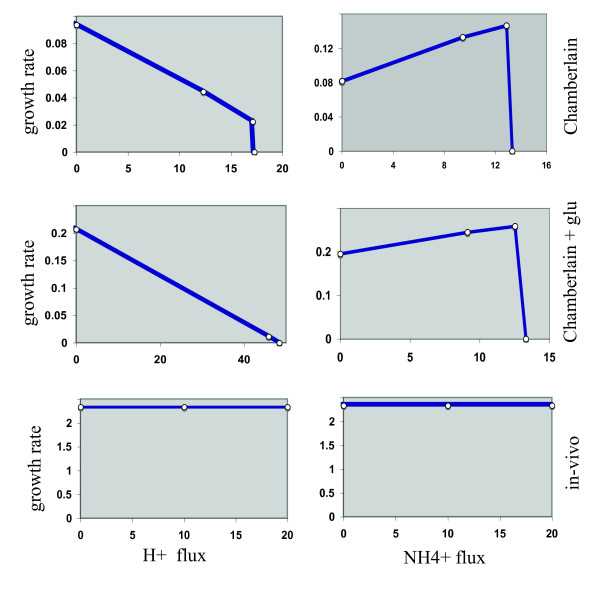
**pH homeostasis predictions by *Francisella *model *i*RS605**. Robustness diagrams depict the effect of proton flux and ammonium ion flux on growth rates of *Francisella *as a function of the environment. Left panels show H+ ion effects, while the panels on the right show NH4+ ion effects. Distinct trajectory types are observed in all media. Media used were Chamberlain minimal medium (Chamberlain), Chamberlain medium supplemented with glucose (Chamberlain +glu), and host cell conditions (as described in Additional File S3).

One particularly striking example of the power of *in-silico *simulation is the investigation of pH homeostasis on cell behavior. In the case of *Francisella *grown in Chamberlain medium the presence of amino acids and their consumption could compensate for excess protons by production and exchange of ammonium (NH4+) ions in the opposite direction. Thus media might serve as pools supplying and dissipating both acidic and basic ions as needed. The calculated ammonium flux out of *i*RS605 is four times that of *Salmonella*. The model was used to correlate the effect of changing H^+ ^and NH4^+ ^ion flux on growth rate in different environments using robustness analysis (Figure 2). The effect on growth rate was highly dependent on the media composition. Completely distinct trajectories were observed in each case.

In the absence of any added carbohydrate source in Chamberlain media, the RDs for NH4^+ ^and H^+ ^ions are complex and consist of multiple trajectories and inflection points. Initially, the H+ ion trajectory has a negative slope and although fluxes move on same time scales, they are in opposite direction. This implies lowering of growth rate as H+ ion flux increases. This is accompanied by a simultaneous increase in NH4^+ ^flux (positive slope on the trajectory I of NH4^+ ^RD) that might neutralize the environment to promote growth. The RDs for both ions show a threshold above which there is a sharp decrease in growth rates. Thus there exists a limiting concentration of these ions that results in a buffered environment ideally suited for growth of *Francisella*. In the presence of glucose (2 mg/ml) in Chamberlain media both NH4^+ ^and H^+ ^ions lower the growth rate gradually. During infection of host cells the growth seems to be independent of the NH4^+^or H^+ ^ion fluxes. This type of analysis demonstrates the power of our systems approach to generate important new hypotheses that can then be addressed experimentally.

### Metabolic profiling of *Francisella*

Metabolic profiling of *Francisella *was done to identify carbon and nitrogen sources (C/N) that it can utilize for respiration. Different growth states of *Francisella *were tested to identify changes in metabolic capacity based on age and growth cycle. Cells were pre-cultured on rich chocolate agar plates to confluence and minimal Chamberlain media with glucose to exponential, mid-log, and stationary phase.

**Figure 3:**
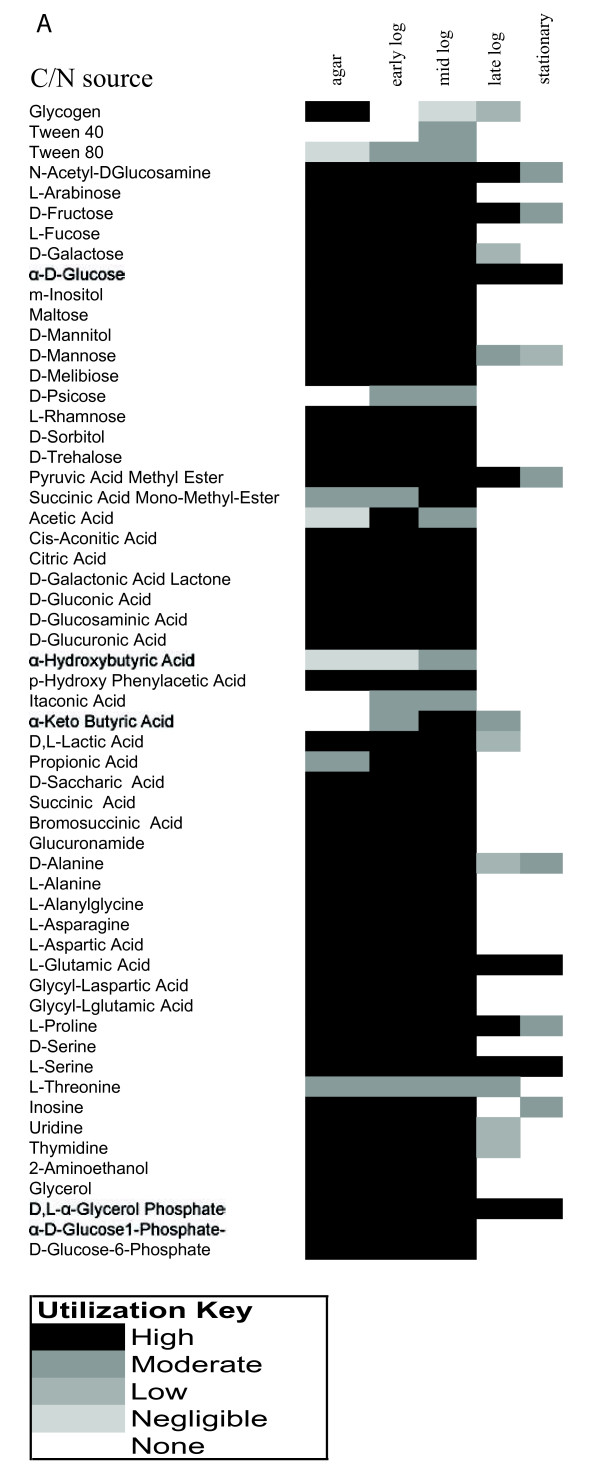
**Metabolic profiling of *Francisella *in different growth phases**. Bacteria were grown overnight on agar or in Chamberlain medium supplemented with glucose to early, mid, or logarithmic growth phase or to stationary phase. Bacteria were harvested and their utilization of various carbon or nitrogen (C/N) sources tested as described in Material and Methods.

Cells in early exponential phase or chocolate agar grown cells were able to utilize a vast array of C/N sources (Figure 3). 60% (57/96) of substrates were utilized which is similar to that observed for several other isolates of *Francisella *as reported in literature [39]. Culturing on chocolate agar reduced the ability to utilize 22% of the carboxylic acids utilized otherwise (Figure 3). Cells in stationary phase lost the ability to respire on most C/N sources (Figure 3). Irrespective of the growth phase they are in, *Francisella *cells can utilize 10 compounds including some hexoses and amino acids completely and 8 compounds including glycoconjugate and pyrimidine salvage intermediates partially for respiration (Figure 3). Although the model could predict steady-state growth accurately for about 50% cases, it currently cannot predict the switch in carbon utilization patterns due to growth phase induced by regulatory effects.

### Differential effect of carbon sources on bacterial growth

**Figure 4:**
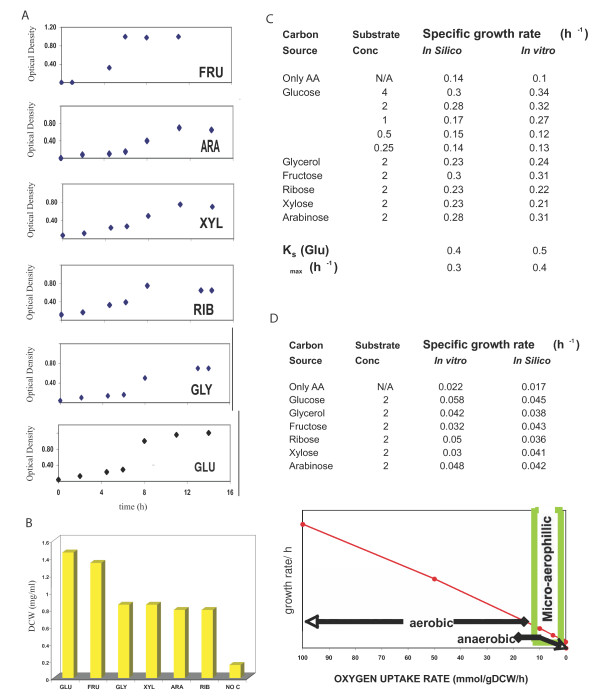
**Experimental and in silico growth characteristics of *Francisella *on carbohydrates**. A. Growth curves of *Francisella *follow monod kinetics. Cells were grown in Chamberlain medium supplemented with the respective carbon source as indicated (FRU; fructose, ARA: arabinose, XYL: xylose, RIB: ribose, GLY: glycerol, GLU: glucose). Optical density (600 nm) was measured every 120 minutes. B. Final biomass yields on different carbon sources. Cells were grown in Chamberlain medium supplemented with carbon sources as in A. Yield is expressed in dry cell weight (DCW). C. Comparison of growth rates in Chamberlain media supplemented with carbon sources as indicated were determined experimentally and calculated in silico under aerobic conditions. Specific growth rates on glucose were used to determine the Um and Ks values. D. Growth rates in Chamberlain medium supplemented with carbon sources as indicated were determined experimentally and calculated in silico under microaerobic conditions. Robustness analysis using *i*RS605 depicts aerobicity based on ranges of oxygen uptake rates and the corresponding effect on growth.

Chamberlain's chemically defined medium with the addition of different sugars was used to experimentally determine the growth and metabolism of *Francisella*. The same media composition was also used to compute the state of *Francisella *cells *in silico *for validation and predictive purposes. Considerable growth was observed on all carbon sources tested as seen in the growth curves (Figure 4A). Growth followed Monod kinetics on all investigated carbohydrates. *F. tularensis *was also able to grow in Chamberlain media on amino acids without an additional carbohydrate source. Increasing the amino acid concentration increased the growth rates as has been reported [40,41]. However, the specific growth rates were slower and the final biomass concentration was much lower (Figure 4B). When we studied the effect of the concentration of glucose on specific growth rates we found an increase in growth rates with increase in glucose concentration with a sudden jump at 1 mg/ml (0.1% wt/vol), the physiological concentration of glucose). Growth on the selected hexoses glucose and fructose allowed similar uptake and growth rates, while the pentoses xylose and ribose resulted in lower growth rates and biomass yields. HPLC analysis of the spent media showed complete consumption of all amino acids added to the medium (data not shown). This confirmed an earlier prediction of the model that amino acids can fulfill the bulk requirement for growth, although additional carbohydrates are needed to achieve better biomass yields. Using FBA we were able to predict the experimentally determined growth rates accurately (Figure 4C). The corresponding uptake rates of all the compounds that define the media composition are represented as exchange fluxes *in silico*. The saturation constant (K_s _= 0.5 mg/ml) and maximum growth rate (μ_max_0.4 hr-1) on glucose were estimated using the experimental data. Using the model to vary initial uptake rates of glucose and to estimate growth rate resulted in a good correlation between experimental and *in silico *results.

Growth under microaerophillic conditions resulted in specific growth rates lower by an order of magnitude (Figure 4D). Oxygen conditions are simulated by specifying a value for the uptake rates of oxygen (defined as exchange flux EX_O2 in the model; see constraints in Additional File S1). The model also predicts an order of magnitude decrease in growth rate on decreasing the oxygen uptake rates (OUR) from 100 to 0.1 mmol/gDCW/hr on all the carbohydrates tested. Our values of less than 4 mmol/gDCW/hr have been observed for several organisms [42,43] under microaerobic conditions. Anaerobic conditions were simulated using a zero oxygen uptake rate.

### Gene expression profiling

The global metabolite pool in the macrophage cytosol defines the substrate availability and nutrient *milieu *for *Francisella*. Variations in gene expression of metabolic pathway genes reflect changes in components of this pool as the pathogen adapts to the host environment. We selected a core set of 69 metabolic genes for quantitative analysis (Additional File S6), which included genes that may be required for adaptation to the host cell by driving metabolism, energy and pH homeostasis. These genes included some house-keeping genes and genes with putative predicted function.

**Figure 5:**
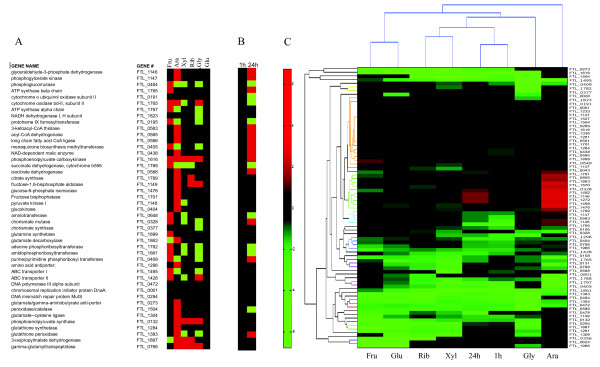
**Selected metabolic transcriptome of *Francisella *in varying environments**. A. Bacteria were grown in Chamberlain medium supplemented with carbon sources as indicated (Fru: Fructose, Ara: Arabinose, Xyl: Xylose, Rib: Ribose, Gly: Glycerol, Glu: Glucose, C: Chamberlain media only). Expression of 75 selected metabolic genes was measured using RT-PCR and GeXP (described in detail in Materials and Methods). Data were normalized to the house-keeping gene FTL_001 and are presented relative to expression in glucose media. B. Macrophages were infected with *Francisella *for 1 h and for 24 h. Expression of selected metabolic genes was quantitatively measured as described in A. Data were normalized to the house-keeping gene FTL_001 and are presented relative to gene expression in macrophage 1 h after infection. C. Composite Heat Map of the selected *Francisella *metabolic transcriptome. Data from A and B were clustered based on environments and functional categories of the genes.

Initially we analyzed the expression of selected metabolic genes during growth under defined culture conditions (Figure 5A) in order to gain insight into the use of metabolic pathways that might correlate to *Francisella*'s intracellular growth. Gene expression was measured quantitatively by using the Genomelab Expression profiler (GeXP) as described in Methods. The media and other physical parameters were kept identical except for carbohydrates added to the medium. During growth on all carbohydrates, the citrate synthase gene, glucose-6-p isomerase, NAD dependent malic gene, pyruvate kinase, isocitrate dehydrogenase, NADH dehydrogenase, chorismate synthase, fructose 1,6 bisphosphatase, glucose kinase, purine/pyrimidine phosphotransferase, glutamine synthase were all active indicating similar metabolic function and up-regulation of central metabolic, amino acid, and nucleotide pathways. However, some of the specific substrate utilizing, amino acid, nucleotide metabolism, and transport genes had different expression profiles, depending on the added carbon source. In the case of growth on pentoses and glycerol, glycolysis and TCA cycle genes (phosphoglucomutase, succinate dehydrogenase, bdII cytochrome oxidase and others) were low (two-fold) compared to growth on hexoses. During growth on pentoses and glycerol, the expression level of the glycine cleavage system (H-protein) was 2-fold that found for hexoses. There was also a 2-fold increase in PEP carboxylase (converts PEP to oxaloacetate) expression. Relatively higher rates (2 fold increase) of aromatic amino acid synthesis (shikimate kinase, anthranilate synthase) also took place when *Francisella *was growing at the expense of fructose and glycerol as carbon-sources. PRPP synthesis was similar in all cases, suggesting similar rates of nucleotide biosynthesis. However, during growth on glycerol adenine phosphoribosyl transferase was very low, suggesting the de-repression by FBP. On comparison with the data where no carbohydrate was added to Chamberlain media, amino acid pathway genes were expressed at higher levels than genes involved in the metabolism of fatty acid oxidation (FAX), oxidative stress response (OX), and gluconeogenesis (GNS).

### Gene expression profiling during *Francisella *infection

We then measured gene expression of selected metabolic genes in *Francisella *recovered from macrophages after one or twenty-four hours of infection (Figure 5B) and compared those to the profiles obtained when *Francisella *was grown in defined culture media.

Several genes of the citric acid cycle were expressed, including succinate dehydrogenase and cytochrome bdII. The data showed a glutamate decarboxylase mediated pH homeostasis in *Francisella *within an hour after infection. ATP synthesis and simultaneous proton exchange was mediated by F1F0 ATP synthase (increased expression) and was required for acid resistance and is critical to survival in the host cell. The pathogen respires oxidatively in the initial stages as indicated by increased ubiquinol oxidase activity. Expression of ubiquinone/menaquinone biosynthesis gene in later stages was detected, suggesting a switch to more microaerophillic environments. Co-expression of glycolysis/gluconeogenesis (phosphoglycerate kinase and glyceraldehyde phosphate dehydrogenase) and fatty acid oxidation genes at this time indicated the switch in carbon source utilization for growth and survival in the macrophage. The gene encoding PEP carboxykinase was highly expressed at 24 hr post infection. Our data also show the utilization of fatty acids as growth substrates, similar to what has been observed for *Mycobacteria *[44]. Several genes involved in the beta-oxidation of fatty acids were seen to be upregulated including a ligase that has been implicated in import of exogenous fatty acids. This is not surprising since fatty acids are the preferentially utilized gluconeogenic substrates during carbohydrate limiting conditions. Anaplerosis seems to be mediated by PEP carboxykinase, which was upregulated in *Francisella*, and also by increased expression of several other genes involved in oxaloacetate synthesis. Detection of catalase and peroxidase early on in *Francisella *suggests a redox active phenotype for the macrophage. Amido ribosyltransferase (AMPRT), which commits pentose phosphate pathway metabolite PRPP to nucleotide biosynthesis, had increased mRNA abundance but there were lowered transcript levels for adenine phosphoribosyltransferase (APRT) at 24 hours post infection. This might result in AICAR accumulation, which is an allosteric regulator for many pathways. These data demonstrate the metabolic adaptations *Francisella *undergoes when replicating in macrophages.

We also observed upregulation of amino acid genes (anthranilate synthase I, chorismate mutase II), sugar metabolism genes (glyceraldehyde-3-phosphate dehydrogenase, phosphoglycerate kinase) and genes related to fatty acid metabolism (phospholipase D and acid phosphatase precursor). This suggests a possible intra-macrophage environment for pathogen metabolism during infection. The up-regulation of both acetyl-coA carboxylase subunits indicates regulation of fatty acid oxidation by synthesis of malonyl-coA. The down-regulation of PEP carboxykinase indicates that there may be other mechanisms of mediating anaplerosis during infection.

Figure 5C summarizes the patterns of gene expression as a composite heat map for carbon source assimilation and energy production leading to growth of *Francisella *in the macrophage and in vitro. Hierarchical clustering analysis (based on Pearson coefficients) showed similarity when *Francisella *was grown on the pentoses (xylose and ribose) or on the hexoses (fructose and glucose) studied. Expression profiles on glycerol resembled that on pentoses. Gene expression levels during growth on arabinose were distinctly different. Gene expression profiles during infection resembled those during growth on defined media with pentoses as carbon sources. Clustering based on genes also showed that genes were expressed similarly based on functional category.

### Gene expression profiles and robustness of *iRS*605

**Figure 6:**
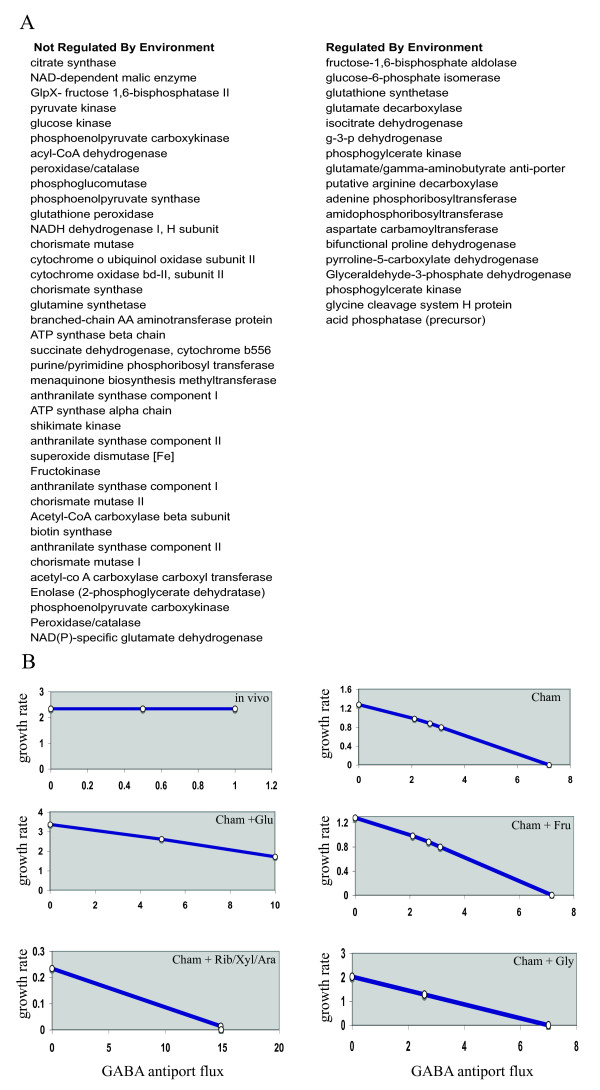
**Metabolic transcriptome and robustness of *Francisella *network**. A. List of genes in the measured metabolic transcriptome that affect growth rate as a function of the environment. B. Representative robustness diagrams for one of the genes listed in A, the glutamate/GABA antiporter involved in glutamate mediated acid resistance. Changing the carbon source in chamberlain minimal media changes how the transport of GABA and glutamate influence of growth rate distinctly.

The model predictions of robustness analysis matched the trends in differential gene expression both *in vivo *and *in vitro*. Among the genes whose expression levels do not affect the growth differently based on environment, are the nodes of metabolic network that are highly connected, like ATP synthase and NADH dehydrogenase (Figure 6A). The reactions catalyzed by the genes whose expression levels affect growth rate (regulated by environment) and those that are not regulated are listed in Figure 6A. The genes whose level of expression affect the growth rate include genes of core central metabolism including nucleotide, acid resistance, carbohydrate functional classes. Figure 6B shows the effect of varying the effect of one such gene involved in Glutamate/GABA antiport on growth in varying environments. The growth rate and GABA antiport flux are separated by different time scales both in vivo and in vitro. *In vitro *(Chamberlain media) the addition of a carbohydrate changed the robustness diagram trajectory. If the flux through the GABA/Glutamate antiporter is equated to the observed mRNA abundance *in vitro*, the growth rate is in the maximum range *in silico*, indicating the time of harvest in exponential growth. The homolog of this gene in *E.coli *forms a part of the glutamate based acid resistance system that protects *E.coli *from the deleterious effects of high proton concentration environments [45].

## Discussion

Metabolism of the pathogen during infection of its host is one of the most fundamental aspects of the host-pathogen relationship. Emphasis on mechanisms and survival strategies aimed at evading the immune responses of the host often ignore that the host cell determines the habitat for the pathogen by limiting concentrations of certain nutrients and other essential metabolic factors. The pathogenicity of *F. tularensis *has been linked to the ability of the pathogen to escape from the phagosome and replicate in the cytosol [46]. Little is known about central metabolic, amino acid, fatty acid, and nucleotide pathways used by *F. tularensis *during infection. This is the first comprehensive study that uses a systems biology approach to define the metabolic capacity of an experimentally ill-characterized pathogen, *F. tularensis*, in multiple environments including the macrophage cell. In this study we designed an *in silico *strain *i*RS605 of *Francisella tularensis *LVS and used it to probe the functional state of the cell.

*Francisella *represents a pathogen with limited experimental datasets and has recently garnered increased attention because of its potential as a biothreat agent. Our systems approach presented here illustrates how constraints-based computational analysis can be used under these circumstances in an efficient way to provide an analysis of the pathogen's metabolic potential during infection and provide a framework for integration of high-throughput datasets. Such an approach is an important testing ground when novel therapeutics or vaccines have to be developed for an emerging pathogen for which initially only high-throughput data such as genomic and transcriptomic data are available.

*Francisella *has a reduced genome although about a quarter of its genome codes for metabolic functions. Only the penultimate or final steps of many metabolic pathways, such as those of amino acid biosynthesis, are maintained in the pathogen. It has a broad range of host specificity, which suggests that its metabolism is specifically tailored to exploit conserved eukaryotic metabolic niches. Thus there may have been a certain selection pressure during the evolution of *Francisella *to select or preserve enzymes with a broader specificity and subsequently reduce genome size and still be an inimitable intracellular pathogen. This is supported by flux balance analysis of *i*RS605, which demonstrates very stringent growth requirements, and by in silico gene deletion analysis, which predicts a high number of 105 condition-independent single essential genes.

Experimental single gene deletion assays have become a principal established tool for studying cell behaviour [5,47,18]. Such experiments, however, are not trivial for class A biothreat agents or for uncharacterized novel pathogens, for which genetic tools and suitable screening assays are not readily available. At the same time, gene deletion data are essential for understanding pathogenesis and, more importantly, for identifying potential targets for antibacterial intervention. Our studies here demonstrate how a genome-scale metabolic reconstruction can very efficiently generate a list of lethal single gene deletions using appropriate constraints. The distribution of essential genes into functional categories shows that most belong to amino acid (22%) and nucleotide (21%) metabolism. The other functional categories that feature in this list include fatty acid/lipid metabolism (12%), cell wall synthesis (19%), cofactor biosynthesis (18%), and extracellular transporters (4%). Only 4% of genes involved in carbohydrate metabolism (all part of the reductive pentose phosphate pathway branch) were identified as essential for the survival of *Francisella*. The significance of amino acid metabolism is also supported by our experimental data that demonstrate that all amino acids in Chamberlain media are consumed in the presence of a carbon source. Asparagine, tryptophan and glutamine synthase genes are essential probably because only their precursors are available to *Francisella *in the host cell. Amino acid decarboxylases may coordinate pH homeostasis along with their corresponding antiporter. Nucleotide metabolism genes are indeed critical to the survival of the pathogen with PRPP being supplied by the pentose phosphate pathway. The production of several intermediates in purine and pyrimidine synthesis is essential and suggests a role as substrates for other networked pathways. Interconversion of nucleotide kinases is important since all the four nucleotide kinases are predicted to be essential. Several fatty acid genes are also essential and suggest the use of fatty acids as gluconeogenic substrates. Combined with limited validation by transcriptomic profiling as presented in this study this approach can thus generate testable hypotheses. These will have to be validated by further experimentation, but our approach significantly reduces the possible solution space. We have also illustrated this approach by highlighting how the *in silico *analysis of pH homeostasis affects the metabolism and subsequently growth of *Francisella*, which correlates well with observed experimental results.

**Figure 7:**
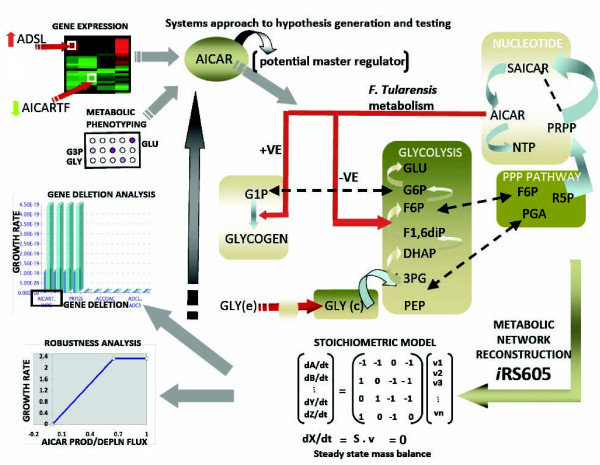
**Systems Biology approach to novel non intuitive hypothesis generation and testing**. Identification of AICAR as a potential master regulator in infection as result of combining multiple diverse data types in the context of a stoichiometric model. FBA can be used on the derived model for *Francisella *to calculate the functional state and identify AICAR as key node.

*In silico *analysis of iRS605 can be used to identify nodes in metabolic networks that act as potential controllers of overall metabolism. One such example is AICAR. The adenylyosuccinate lyase gene that catalyzes its formation, is a condition independent lethal gene, essential for survival. Robustness analysis showed that flux through the adenylosuccinate lyase and AICAR formyltransferase genes need to be in equilibrium for optimum growth (production and consumption of AICAR must be at steady state). AICAR is a known allosteric regulator of several enzymes in carbohydrate and amino acid metabolic pathways [48]. AICAR is also known to stimulate beta-oxidation and drive the equilibrium of FBP towards gluconeogenesis. Fatty acid utilization for gluconeogenesis during infection is also supported by increased AICAR synthesis, which is known to stimulate beta-oxidation. mRNA transcript data in Chamberlain media supplemented with glycerol and glucose also suggest an accumulation of AICAR as a regulator of fructose bis phosphatase *(fbp) *gene in *Francisella*. Thus, the predicted accumulation of AICAR in the macrophage suggests a role as a potential master regulator during infection. Such hypotheses are not readily intuitive and further demonstrate the usefulness of a systems biology approach (Figure 7).

Our *in silico *analysis was able to demonstrate that *Francisella *undergoes changes of its metabolism when it enters its host. Flux variability analysis determines all possible alternate routes for growth of a bacterium that are necessary under specific conditions. *i*RS605 would thus be able to achieve this objective with a smaller reactome in nutrient rich conditions than in a minimal medium *in silico*. The common reactome of *Francisella *during infection and during growth on Chamberlain medium with glucose (Additional File S5) is comprised of genes required for growth and macromolecule formation including nucleotides and fatty acids. The microbe tends to utilize preferentially specific amino acids for energy and fatty acids as gluconeogenic substrates rather than relying on carbohydrate sources like glucose and fructose during infection. These predictions have been validated by selective quantitative gene expression profiling as discussed earlier. The significance of these findings remains to be further elucidated, especially in conjunction with further research as to what particular metabolic niche the host provides and how it might deal with intracellular pathogens by restricting the availability of certain nutrients. Previous metabolic analysis of the model pathogen *Salmonella *[5], which displays greater variability of its metabolic pathways during intracellular growth, revealed alternate functional pathways that are operational during infection. Models and data for both organisms, however, demonstrate a robust limited set of reactions required for intracellular proliferation. Thus, a systems biology approach as presented here reduces the possible solution space for novel antibacterial targets.

Constraints-based modeling approaches have now been used for a variety of prokaryotic and eukaryotic organisms, but usually for those with a significant body of experimental data. In most of these cases the modeling efforts could predict experimental findings with some accuracy and correlated with prevailing hypotheses about pathogenesis. In *Mycobacterium tuberculosis *constraints-based modeling was also used to predict potential targets for antibacterial therapy in the mycolic acid pathways, which has always been the main attention of antituberculous research [49]. The challenge in pathogens which have not been extensively studied but which may gain rapid notoriety due to intentional release into the population or due to sudden unanticipated epidemic spread, is the rapid identification of Achilles heels that can be exploited for intervention. Our studies here demonstrate that constraints-based modeling might significantly aid such efforts.

## Conclusions

We have presented here the reconstruction of a genome-scale metabolic model for the biothreat agent *Francisella tularensis *(*i*RS605). This model has been validated by legacy data, experimental phenotypic arrays, selective determination of metabolites, and growth profiles on defined minimal media. We demonstrated an accuracy of almost 80% in predicting growth and virulence phenotypes.

Analysis of the model showed significant changes of metabolism during Francisella's intracellular growth in its host cell, the macrophage. This was evident in a switch from oxidative metabolism (TCA cycle) in the initial stages of infection to glycolysis, fatty acid oxidation, and gluconeogenesis during the later stages. Computational analysis also demonstrated a limited set of metabolic genes that are likely to be operational during infection. These findings were corroborated by quantitative gene-expression profiling of selected genes that code for key metabolic enzymes. Prediction of synthetic lethals also identified a set of potential drug targets.

We have thus demonstrated how such an integrated systems approach can be used for pathogens with limited extended datasets to elucidate key metabolic processes during pathogenesis and identify potential novel drug targets.

## Methods

### Metabolic Reconstruction

Reconstruction and model development of the metabolic network followed established methods [5,3]. The reconstruction process and the contents of the *i*RS605 are summarized in Additional File S1. The reconstruction process was done using SimPheny™ (Genomatica, Inc., San Diego, CA), a platform for cellular model development, which includes an interface for references, annotation, and confidence levels of annotation. The sequence based genome annotation of *F. tularensis subsp. holarctica *( GenBank Accesion AM233362.1) was downloaded and served as the framework for the initial draft of reconstruction. Charge and elementally balanced reactions were added individually based on this annotation and legacy data when available. Biohealthbase and FranTCyc were used as ancillary tools. Following the initial reconstruction, the gaps were evaluated individually by searching for direct evidence in the literature for their metabolism. Due to the relative paucity of literature for *F. tularensis*, many of these gaps could not be filled. Legacy data in the form of primary articles, review articles, and textbooks were employed in addition to the database resources during the reconstruction and model building phases. If an annotation was made to an ORF that supplemented the annotation found in the genome file or that led to additional functional assignments, this was noted in the reaction database for the model in Simpheny. Also, high sequence homology of an ORF in the LVS strain, compared to genes in other species of francisella with strong experimental evidence were included in the model. A comprehensive map of *i*RS605 metabolic network was visualized by creating a map of the network organized by lumped subsystems of metabolism. After the debugging process, when the cell could grow (i.e. produce biomass) on different media, the remaining intracellular gaps were evaluated by searching the literature for evidence of metabolic reactions involving that particular metabolite in different species of the bacterium. If no evidence of transport or biochemical transformations of the metabolite in *F. tularensis *was found, no additional reactions or transporters were added.

### Biomass Composition and Reaction

A reaction that represents biomass production is included in the model to account for the drain of precursors and macromolecular building blocks into biomass. Biomass was represented as a linear combination of all the macromolecular components (lipid, glycogen, lipopolysaccharide, and peptidoglycan) or monomers of macromolecules (amino acids and nucleotides). Amino acid and fatty acid composition were experimentally measured (data not shown) using standard methods [5]. The relative fatty acid composition was used to specify the average fatty acid content on the phospholipds and diacylglyerol. *F. tularensis *data from the literature was then used to determine the amount of lipids (and their phospholipid composition), lipopolysaccharide, peptidoglycan, and glycogen. The stoichiometric ratios of all the components in biomass are given in the non-gene associated reaction in Additional Files.

The growth associated ATP maintenance was calculated using data from Figure 4 and Flux Balance Analaysis (FBA). The measured fluxes were set as constraints and FBA was used to calculate the maximal amount of ATP that could be generated. The resulting ATP production reflects the amount required for growth and maintenance, additional to the ATP required for precursor biosynthesis. When the maximal ATP production rate is plotted against growth rate, the intercept gives the maintenance coefficient (in mmol h^-1 ^gDW^-1^: 95), and the slope gives the amount of ATP (in mmol gDW^-1^) required for assembly. The high ATP value is indicative of less energetically efficient pathway usage. The model will always use the reactions that give the highest P/O ratio. If the organism uses instead, a less energetic enzyme systems, then the ATP terms would be lower because of a forced lower P/O ratio. This is similar to organisms like *Lactobacillus *and *Shewanella *species (Jennifer Reed, unpublished results). The biomass reaction thus represents the weighted combination of components forming the dry weight of the cell and the amount of ATP hydrolysis, needed for energy during growth and cellular maintenance as discussed.

### Constraint-based modeling

The reconstructed metabolic network was represented by a stoichiometric matrix, S (m × n), where m is the number of metabolites and n is the number of reactions. Reactions within the network were mass balanced such that Sv = 0, where v was a steady-state flux vector. Additional constraints (like media composition, gene expression data) on each reaction had the form _i _v_i _ß_i_, where _i _and ß_i _represented the lower and upper limits, respectively. _i _was set to zero for irreversible reactions, whereas ß_i _was set to measured uptake rates for transport reactions or the V_max _of the corresponding enzymes.

The biomass reaction thus represents the weighted combination of components forming the dry weight of the cell and the amount of ATP hydrolysis, needed for energy during growth and cellular maintenance as discussed elsewhere.

### Flux Balance Analysis

Fluxes through metabolic reactions across the network can be calculated using flux balance analysis (FBA). With FBA, the biological system is assumed to be at steady-state so that all intracellular metabolite concentrations and fluxes are assumed to be constant. The steady-state assumption makes it possible to compare the simulation results directly to data obtained from cells growing at a fixed growth rate. FBA is formulated as an optimization problem, where constraints are imposed that limit flux values (steady-state mass balance constraints and upper and lower bounds for fluxes based on thermodynamics and substrate uptake constraints). These constraints define the range of values that fluxes can take. An objective function is also used to compare flux distributions (v) that satisfy all the constraints in order to find optimal flux distributions. Flux through the biomass production reaction has successfully been used as an objective function for *E. coli *(along with other objective functions) [50] and was also used for FBA performed in this study. Thus, FBA predicts an optimal growth yield and a flux distribution(s), which correspond to this maximal growth yield. Alternate optimal solutions exist, and flux variability analysis was additionally used to identify the ranges individual fluxes can take while still achieving the maximal growth yield. Reaction flux, is measured in mmol/h/g (dry weight), and the growth rate was reported in units of 1/h. Ammonia exchange fluxes were estimated using flux balance analysis. The effect of various other parameters on ammonium flux was calculated using robustness analysis. The objective of the model (in silico cell) was growth and the constraints were set to represent the environmental conditions and the flux through the ammonium exchange reaction was calculated using linear optimization at steady state.

### Robustness Analysis

Robustness analysis is performed by varying a particular flux through a pre-defined range and recalculating the objective function. The slope of the curve describes the sensitivity of the objective function on that particular flux (over the specified range of values). The flux through several reactions in the metabolic network was varied and the growth rate was calculated.

### *In silico *constraints and media composition

Two different environments were used to provide constraints for simulations: (i) Chamberlain minimal medium (contains 13 amino acids in addition to salts, phosphate, sulfate, ammonium minerals) with a variable carbon source [11]; (ii) host-cell nutrient environment (representing nutrient conditions inside a host-cell during infection). While the host-cell nutrient environment was difficult to ascertain, it was based on an extensive literature review to identify a possible composition [5]. Details of all simulated media conditions are provided in Additional Files (S3). Additional File S1 shows how media composition is represented as exchange fluxes to use as constraints in the model. For simulation of aerobic growth, the following external metabolites were allowed to freely enter and leave the network: phosphate, ammonium, sulfate, water, oxygen, and proton. All metabolites that were not media components (potential products) were only allowed to leave the system.

### Growth and Infection Experiments

*F. tularensis *LVS strain was used for all experiments. LVS was grown in *Chamberlain *media. *Chamberlain *media was supplemented with 4 mg/ml of the following sugars: glucose, glycerol, fructose, arabinose, ribose, and xylose. All *in vitro *growth experiments were done in an incubator shaker at 37°C and 150 rpm. Samples were withdrawn every 2 hours and the optical density (OD) measured. OD was correlated to dry weight using a previously determined calibration curve (y = 0.74 ×). Samples were further syringe filtered and centrifuged in order to obtain supernatants for HPLC analysis. Infection of the macrophage-like cell line RAW264.7 was performed as described previously [51].

### HPLC Analysis

Organic acid and sugar analysis was done using an ion exchange Aminex 87H^+ ^column (Biorad). A 5 mM Sulfuric acid mobile phase at a flow rate of 0.5 ml/min at 65°C was used for separation. Both UV and RI (refractive index) detector were employed. Small molecules were quantified using previously determined calibration curves.

Amino acid analysis was performed using a Waters AccQTag column. Samples were derivatized using the kit and the protocol recommended to form a 6-aminoquinolyl-N-hydroxysuccinimidyl carbamate derivative of the amino acid. The derivatized amino acids were then separated and detected using the C18 reverse phase AccQ-Tag column, with a flow rate of 1 ml/min at 37°C using a 12.5 mM sodium phosphate: Acetonitrile mobile phase with a previously published gradient profile. UV detection was employed and the amino acids were quantified using previously determined calibration curves.

## Metabolic phenotyping

Metabolic profiling was performed using GN2 plates. (Biolog, Inc). Protocols provided by the manufacturer were followed. For experiments determining the effect of growth state on metabolic phenotype, *Francisella *was harvested at different growth phases. Early exponential phase was defined in *Francisella *by an optical density (OD) of 0.32 AU, while ODs of 0.45 AU and 1.2 AU defined mid logarithmic and stationary phase cells.

### Quantitative Gene expression using GeXP™

Infection of the macrophage like cell line (RAW246.7) was carried out as described [51]. RNA was purified from harvested bacterial cells using the protocol from RNeasy Kit (QIAGEN) and quantitated using UV detection at 260 nm and 280 nm. The GenomeLab™ GeXP Genetic Analysis System (Beckman) was used for quantitative gene expression analysis of the selected metabolic transcriptome of *F. tularensis*. GeXP employs eXpress Profiling (XP-PCR), a patented technology for multiplex gene expression profiling analysis by which up to 30 genes can be easily multiplexed in the same reaction. We used a protocol that comes with GeXP Reagent kits that involved five basic steps: 1) Primer design; 2) cDNAsynthesis; 3) PCR; 4) Separation on the GenomeLab GeXP Genetic Analysis System; 5) Fragment Analysis and Expression Profiling. The data was then analyzed using the GeXP analysis proprietary software (Beckman, Inc.), Excel (Microsoft, Inc.) and Matlab (Mathworks, Inc.). Primers were designed using proprietary software provided by Beckman (Additional File S7).

## Authors' contributions

AR conducted the reconstruction process and performed all the analyses. AR and SS obtained all the experimental data. AR and SD planned and designed the study, analyzed, and interpreted all the data. AR and SD drafted the manuscript. All authors approve the content of this manuscript.
